# Src kinase up-regulates the ERK cascade through inactivation of protein phosphatase 2A following cerebral ischemia

**DOI:** 10.1186/1471-2202-10-74

**Published:** 2009-07-14

**Authors:** Xiaohan Hu, Xiangyang Wu, Jiali Xu, Jin Zhou, Xiao Han, Jun Guo

**Affiliations:** 1 Key Laboratory of Human Functional Genomics of Jiangsu Province, Nanjing Medical University, Nanjing 210029, PR China; 2 Department of Biochemistry, Nanjing Medical University, Nanjing 210029, PR China; 3 Department of General Surgery, The Nanjing First Hospital Affiliated to Nanjing Medical University, Nanjing 210006, PR China; 4 Laboratory Center for Basic Medical Sciences, Nanjing medical university Nanjing, 210029, PR China

## Abstract

**Background:**

The regulation of protein phosphorylation requires a balance in the activity of protein kinases and protein phosphatases. Our previous data indicates that Src can increase ERK activity through Raf kinase in response to ischemic stimuli. This study examined the molecular mechanisms by which Src activates ERK cascade through protein phosphatases following cerebral ischemia.

**Results:**

Ischemia-induced Src activation is followed by phosphorylation of PP2A at Tyr307 leading to its inhibition in the rat hippocampus. SU6656, a Src inhibitor, up-regulates PP2A activity, resulting in a significant decreased activity in ERK and its targets, CREB and ERα. In addition, the PP2A inhibitor, cantharidin, led to an up-regulation of ERK activity and was able to counteract Src inhibition during ischemia.

**Conclusion:**

Src induces up-regulation of ERK activity and its target transcription factors, CREB and ERα, through attenuation of PP2A activity. Therefore, activation of ERK is the result of a crosstalk between two pathways, Raf-dependent positive regulators and PP2A-dependent negative regulators.

## Background

When blood flow is decreased to the brain, cerebral ischemia, a complex signaling network is activated. Excitotoxicity can be induced by an increase in intraneuronal Ca^2+ ^through calcium ion channels, such as the N-methyl-D-aspartic acid (NMDA) receptor, L-type voltage-gated calcium channels (L-VGCC) and IP3 receptor. An influx in intraneuronal Ca^2+ ^is a key mediator in multiple intracellular signaling cascades after ischemia [1]. Extracellular signal – regulated kinase (ERK), a member of the mitogen-activated protein kinase (MAPK) family, is activated in a Ca^2+^-dependent manner in cerebral ischemia [2-4]. ERK is regulated via phosphorylation at various motifs. Previous studies have demonstrated that ERK is activated by various upstream kinases, such as Akt and Src, by the Raf/MEK/ERK signaling cascade [5-7]. In response to ischemic stimuli, active Src kinase activates ERK through Raf phosphorylation at Tyr340/Tyr341 [8]. Src is a non-receptor protein tyrosine kinase (PTK). The Src family of proteins contain a Src homology (SH) 2 domain and SH3 domain, a catalytic domain, and a C-terminal tail. Src maintains basal activity in normal cells by an auto-inhibitory mechanism, whereby Tyr527 in the C-terminal tail is phosphorylated allowing for association with the SH2 domain preventing catalytic activity. Src activation is initiated when Tyr527 is dephosphorylated and, subsequently, autophosphorylated at Tyr-416 [7].

In general, changes in protein phosphorylation require coordinate regulation of protein kinases and protein phosphatases. However, to date, the signaling mechanisms leading to dephosphorylation of ERK resulting in inactivation have not been well defined. Protein phosphotase 2A (PP2A) has been shown to be an ERK phosphatase. Furthermore, it has been suggested that PP2A dephosphorylates critical residues resulting in ERK inactivation [9-11]. PP2A is a Ser/Thr-specific phosphatase composed of two regulatory (A and B) and one catalytic subunit (C) [12]. The catalytic subunit of PP2A (PP2AC) is regulated by phosphorylation at Tyr307 in the conserved C-terminal domain of the catalytic subunit resulting in inactivation of the enzyme [12,13]. Active Src can directly phosphorylate PP2A C at Tyr307 [14] and cerebral ischemia leads to up-regulation of Src activity [15]. However, whether Src kinase induces ERK activation through inhibition of PP2A during cerebral ischemia is unclear.

Following ischemia, the activated ERK cascade regulates gene expression via upregulation of specific transcription factors [16]. One of the upregulated targets is estrogen receptor α (ERα), a neuroprotector. It has been reported that ERα phosphorylation at Ser118 positively regulates its function and that phospho-ERK phosphorylates this residue [17-20]. Another neuroprotective protein induced by ERK activation is cyclic AMP response element-binding protein (CREB). Additionally, ERK phosphorylates CREB at Ser133, which is essential for CREB-mediated effects on transcription [21,22]. Interestingly, this residue has been shown to be dephosphorylated by PP2A [23,24].

Therefore, in the present study, we examine the involvement of PP2A in Src-dependent ERK phosphorylation in the rat hippocampus following ischemia. Through the use of SU6656 (SU), a Src inhibitor, we show that PP2A activity is upregulated, which may, in turn, attenuate ERK activation and its downstream proteins, CREB and ERα, in the post-ischemic hippocampus. Thus, we propose that Src induces ERK activation through downregulation of PP2A activity in cerebral ischemia.

## Results

### Src inhibitor decreases activity of the ERK/CREB and ERα pathways post-ischemia

**Figure 1:**
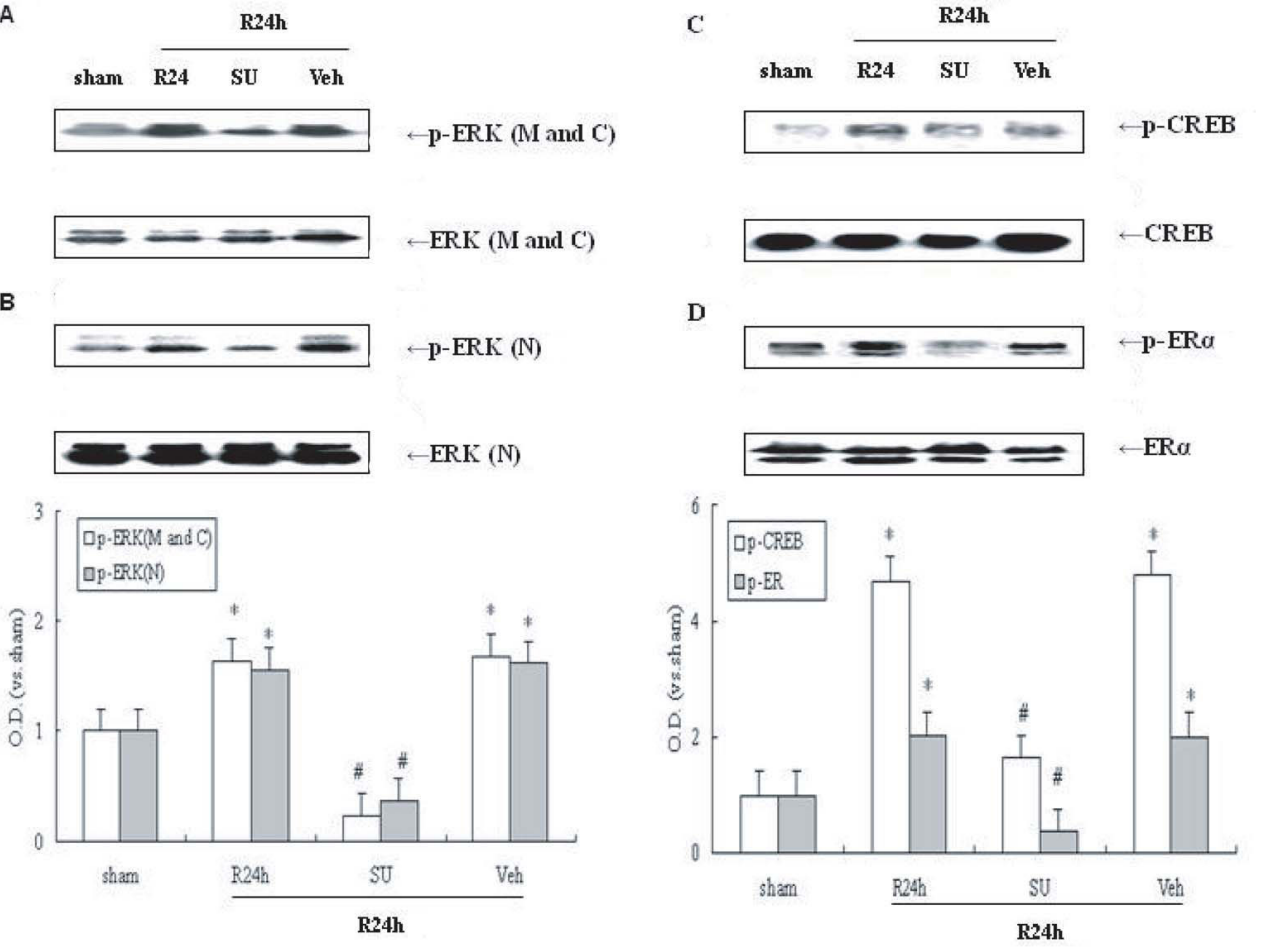
**Western blot analyses of p-ERK, p-CREB and p-ERα protein levels and the effect of Src inhibition on them following cerebral ischemia**. SU6656 (SU) and its vehicle (Veh) were administered 20 min before ischemia (I.c.v.) respectively. 24 h reperfusion after 10 min ischemia was performed. (A) Effect of SU on p-ERK (Thr202/Tyr204) in plasma membrane and cytoplasm (M and C) following cerebral ischemia, (B) Effect of SU on p-ERK in nucleus (N) post-ischemia, (C) Role of SU in p-CREB (Ser118) following post-ischemic reperfusion, (D) Role of SU in p-ERα (Ser133) during reperfusion post-ischemia. Bands were scanned and the optical density (O.D.) is presented as fold-increase compared to sham control levels. Data are expressed as mean ± S.D. (*n *= 4/group), ^#^*P *< 0.05 vs. the respective control group.

ERK can be expressed widely and is found in the cell membrane, cytoplasm, and nucleus [16]. Some previous studies have suggested that the ERK signaling cascade may be up-regulated in a Src-dependent manner following cerebral ischemia [15,25]. Therefore, it was determined if Src kinase regulates ERK activity at different and specific subcellular sites in response to ischemic stimuli. ERK and p-ERK were measured by immunoblot in defined subcellular regions, including the cell membrane, cytoplasm and nucleus. SU6656 (SU, a selective inhibitor of Src) was used to inhibit Src activity. Rats underwent 4-VO and endured 10 min ischemia followed by 24 h reperfusion. Ischemia leads to phosphorylation of ERK during the 24 h reperfusion post-ischemia in the plasma membrane and cytoplasm (M and C) and nucleus (N) (Figure. 1A and 1B, *P *< 0.05). SU effectively attenuated ERK phosphorylation after 24 h reperfusion. In addition, no changes were observed in the total protein levels of ERK. These data indicate that cerebral ischemia results in an increase in ERK activity which is independent of subcellular localization, but dependent on Src activation in the post-ischemic hippocampus.

Activated ERK can facilitate the phosphorylation of a variety of transcription factors phosphorylation involved in gene expression [16]. Two proteins regulated by ERK are CREB and ERα, which are activated by phosphorylation at the Ser133 residue [22] and at the Ser118 site [20], respectively. In this study, CREB and ERα nuclear activity were observed in response to cerebral ischemia. Compared with a sham group, both phospho-CREB and phospho-ERα are increased in the 24 h reperfusion groups (Figure. 1C and 1D; *P *< 0.05) and similar to ischemia-induced ERK activity. To determine whether Src might regulate CREB and ERα activity following ischemia, SU was employed. Levels of p-ERα and p-CREB in the 24 h reperfusion group showed obvious decrease in animals in which SU was administered (Figure. 1C and 1D; *P *< 0.05). These data suggest that Src kinase is required for activation of ERK and, subsequently, ERα and CREB post-ischemic hippocampus.

### Src activation is correlated with an increase in PP2A phosphorylation and inhibition

**Figure 2:**
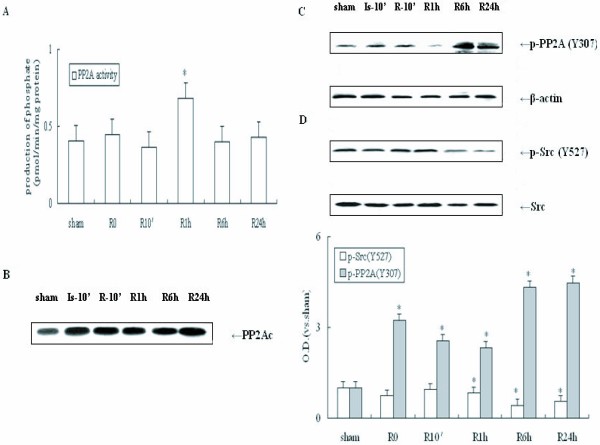
**Western blot analyses of p-Src and p-PP2A C levels following post-ischemic reperfusion**. Samples were taken from the hippocampi of rats subjected to sham, or 0, 10 min, 1 h, 6 h and 24 h reperfusion after 10 min ischemia. PP2A activity was measured using a molybdate dye-based phosphatase assay kit. Sample proteins were identified by special antibodies against p-Src (Tyr527), Src, PP2A C and p-PP2A C (Tyr307). (A) Temporal curve of PP2A activity following post-ischemic reperfusion. (B) Temporal alterations of PP2A C levels during reperfusion post-ischemia. (C) Temporal alterations of p-PP2A C and β-actin levels following post-ischemic reperfusion. (D) Temporal alterations of p-Src and Src protein levels after post-ischemic reperfusion. O.D. is presented as fold-increase compared to sham control levels. Data are expressed as mean ± S.D. (*n *= 4/group), *P < 0.05 *vs*. sham control.

In general, ERK, ERα and CREB phosphorylation are determined by a balance in the activity of upstream kinases and phosphatases. It has been suggested that the Ser/Thr-specific phosphatase, PP2A, might negatively regulate ERK, ERα and CREB activity [26-28]. To examine whether PP2A is involved in the regulation of the Src/ERK pathway post-ischemia, it was first assessed whether ischemia-induced alteration of PP2A activity. All samples were from rats subjected to various reperfusion times (0, 10 min, 1 h, 6 h and 24 h) after 10 min ischemia. Tissue extracts of the hippocampi were processed and assayed using a PP2A activity assay system. The peak of PP2A activity was observed at approximately 1 h of reperfusion (approximately 1.5 times higher than the sham group, Figure. 2A, *P *< 0.05). Sustained inactivation of PP2A activity was observed after 6 h and 24 h of reperfusion and was concomitant with upregulation of the ERK cascade. In addition, no changes were observed in the total protein of PP2A C (Figure. 2B, *P *> 0.05). To confirm inhibition of PP2A activity, immunoblot were performed to assess PP2A phosphorylation at the Tyr307 site in the hippocampus during post-ischemic reperfusion. Hippocampal tissue extracts were prepared as previously described for Figure. 1. As shown in Figure. 2C, ischemia resulted in marked dephosphorylation of PP2A at Tyr307 after 1 h reperfusion, indicating that PP2A activation was induced by ischemia. However, significant phosphorylation of PP2A at Tyr307 was observed after 6 h reperfusion (*P *< 0.05), indicating sustained inactivation of PP2A. Active Src kinase directly phosphorylates PP2A at Tyr307 [12,29]. Therefore, it was determined whether Src is required for inactivation of PP2A in cerebral ischemia. Induction of cerebral ischemia results in dephosphorylation of Src at Tyr527 increasing its activity at by 6 h reperfusion (Figure. 2D, *P *< 0.05). Therefore, ischemia-induced Src activation is accompanied by PP2A inhibition. No changes were observed in the total protein of Src and PP2A in each group (Figure 2B–D, *P *> 0.05). *β*-actin protein levels, used as a control, also remained stable in each group (Figure 2C, *P *> 0.05).

### Inhibition of Src activity results in PP2A increased activity in response to cerebral ischemia

**Figure 3:**
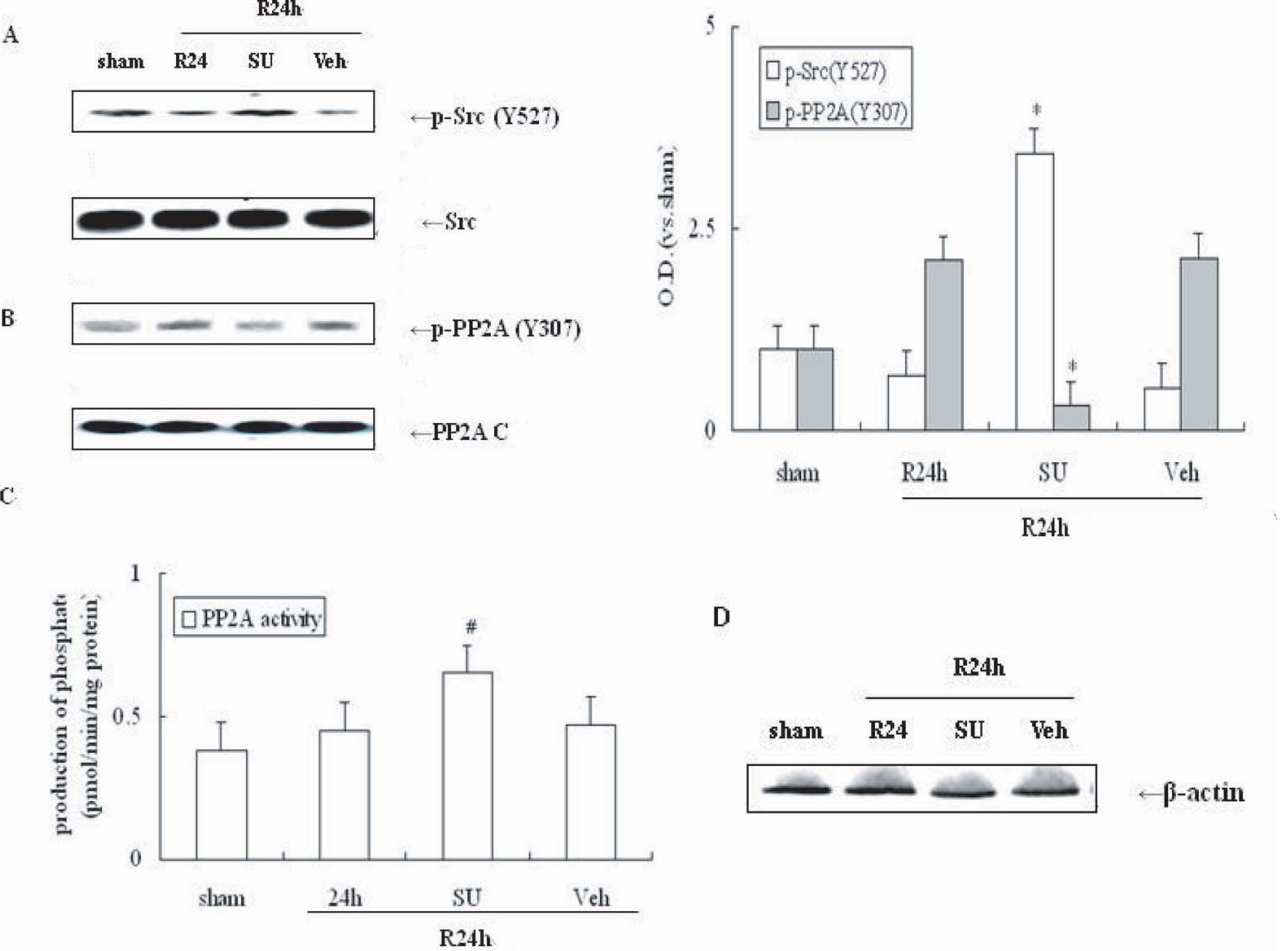
**Effects of SU on the Src/PP2A C signaling pathways in response to cerebral ischemia**. SU or its Veh was administered into the left intracerebral ventricle 20 min prior to occlusion. 24 h post-ischemic reperfusion was performed. Proteins were measured using the same antibodies mentioned in Figure 2. (A) Determination of p-Src levels with and without SU during 24 h reperfusion following 10 min ischemia. (B) Determination of p-PP2A C levels with and without SU following cerebral ischemia. (C) Alteration of PP2A C activity following post-ischemic reperfusion. (D) β-actin with and without SU during reperfusion post-ischemia. O.D. is presented as fold-increase compared to sham control levels. Data are expressed as mean ± S.D. (*n *= 4/group), *P < 0.05 *vs*. sham control.

The results presented above suggest that activated Src kinase likely regulates PP2A activity through phosphorylation at Tyr307 following cerebral ischemia. In order to determine whether Src activation is required for inactivation of PP2A in cerebral ischemia SU was employed. Immunoblot was performed to assess p-Src and p-PP2A levels in the hippocampi of ischemic animals. Rats underwent 4-VO and endured 10-min ischemia followed by 24 h reperfusion. As shown in Figure 3A, SU produced an additive increase in Src phosphorylation at Tyr527 site resulting in a decrease in Src activity, since Tyr527 is an inhibitive site of Src. In contrast, SU also inhibited PP2A phosphorylation at Tyr307 after 24 h reperfusion (Figure 3B, *P *< 0.05). These data indicate that Src activation is required for PP2A phosphorylation following ischemia. These same tissue extracts were processed and assayed using a PP2A activity assay system. As expected, the PP2A activity was higher in samples treated with SU compared with the sham group (Figure 3C, *P *< 0.05). Furthermore, the total protein levels of Src, PP2A c and *β*-actin remained unchanged in each group (Figure 3A–3D, *P *> 0.05). These data show that Src activation is required for PP2A inhibition following cerebral ischemia.

### Inhibition of PP2A compensates for inhibition of Src allowing for upregulation of ERK/CREB and ERα in the presence of SU

**Figure 4:**
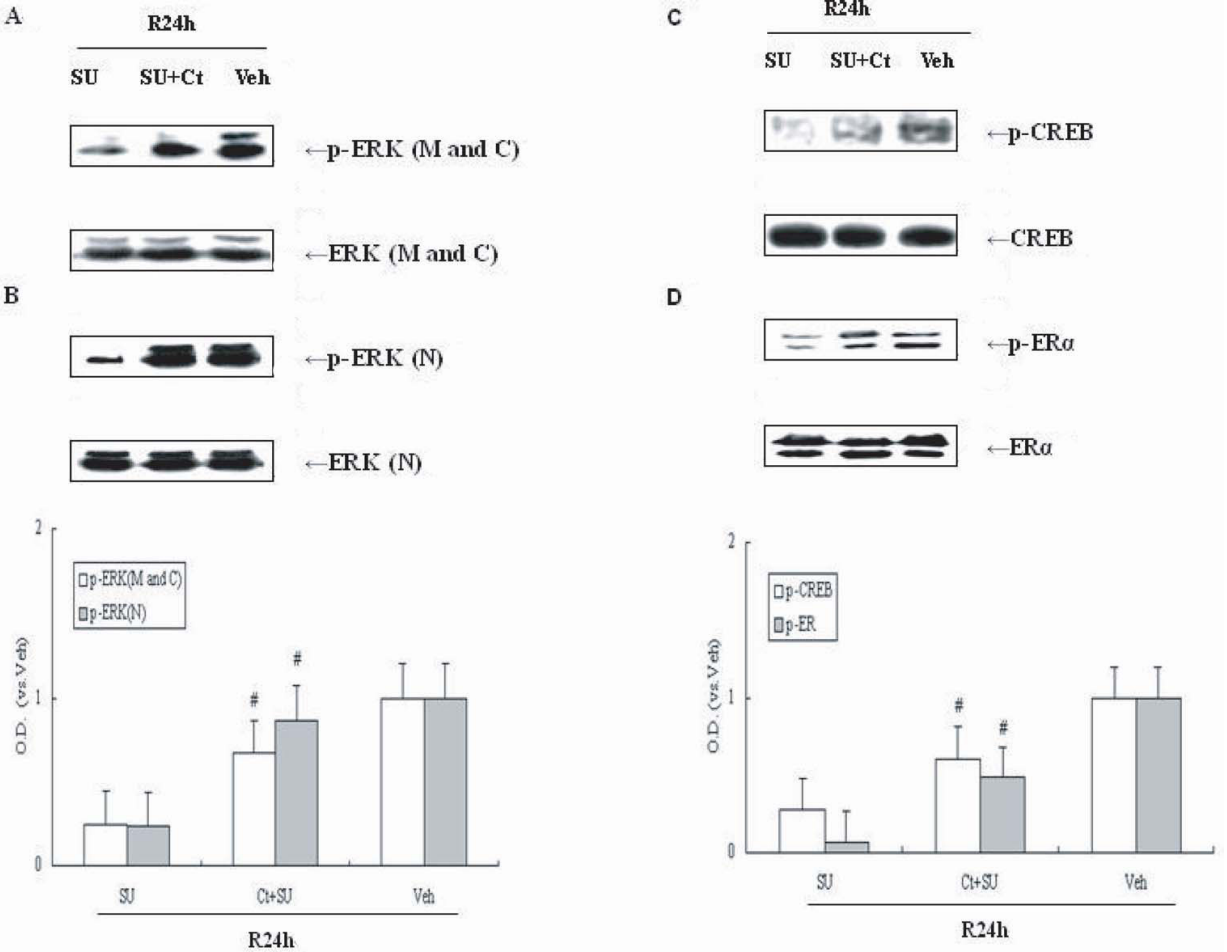
**Western blot analyses of p-ERK, p-CREB and p-ERα protein levels and the effects of Ct and (or) SU on them in cerebral ischemia**. Cantharidin (Ct) and (or) SU were administered at 24 h reperfusion after ischemia. (A) Effect of SU and co-administration with Ct and SU on p-ERK in plasma membrane and cytoplasm (M and C) following post-ischemic reperfusion, (B) Effect of SU and co-administration with Ct and SU on p – ERK in nucleus (N), (C) Effect of SU and co-administration with Ct and SU on p – CREB following post-ischemic reperfusion, (D) Effect of SU and co-administration with Ct on p – ERα following post-ischemic reperfusion. The results are expressed as fold changes versus the sham controls. O.D. is presented as fold – increase compared to Veh controls. Data are expressed as mean ± S.D. (*n *= 4/group), ^#^*P *< 0.05 vs. the respective SU administration group.

Although activated Src kinase decreases PP2A activity after cerebral ischemia, it is not known whether PP2A is involved in the Src/ERK cascade following cerebral ischemia. SU and Cantharidin (Ct), a PP2A inhibitor, was administered prior to ischemia (i.c.v.), and ERK and p-ERK protein were examined in the cell membrane, cytoplasm and nucleus of post-ischemic hippocampi. As shown in Figure 4A, samples from animals treated with both Ct and SU had significantly higher ERK phosphorylation compared with those samples treated with SU only (*P *< 0.05). These data demonstrate that ischemia induces Src activation leading to inhibition of PP2A activity resulting in ERK activation.

PP2A is also able to dephosphorylate ERK targets, CREB and ERα. To further assess the role of PP2A in regulation of signaling cascades during cerebral ischemia, intranuclear CREB and ERα immunoblot assays were performed using specific phosphorylation antibodies. Compared to samples treated with SU only, both ERα and CREB phosphorylations are increased in the Ct and SU co-administration samples (P < 0.05; Figure 1B). These data suggest that Src is required for up-regulation of CREB and ERα pathway through inhibition of PP2A activity. Total protein of ERK, CREB and ERα in each group remained unchanged (Figure 4A–B, *P *> 0.05).

## Discussion

**Figure 5:**
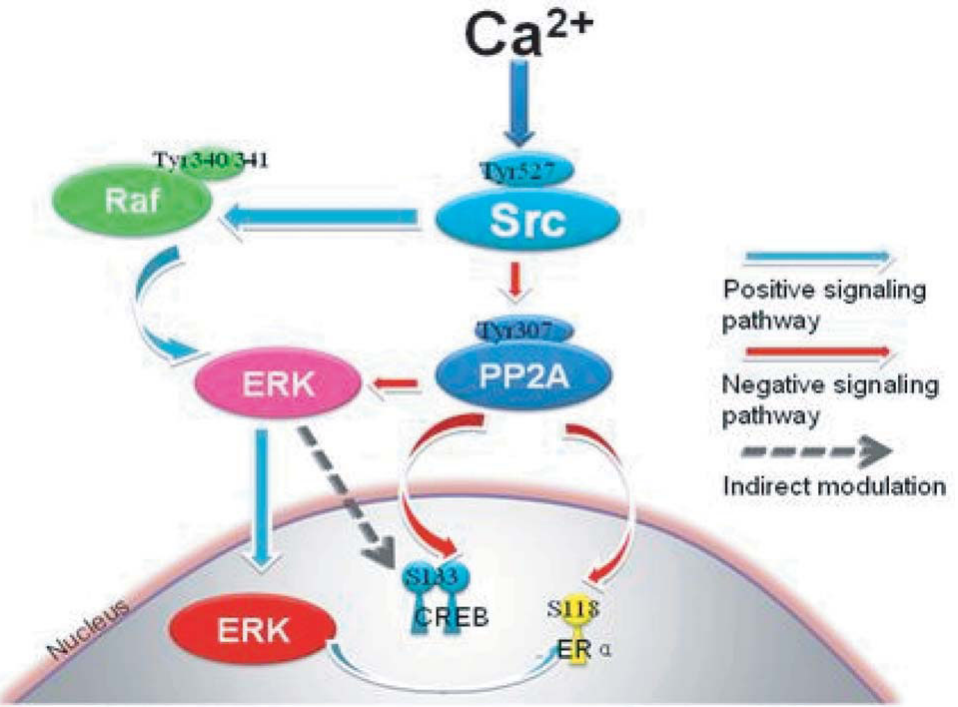
**Crosstalk between Raf-dependent, positively regulated signal cascades and a PP2A-dependent negative signaling pathway in cerebral ischemia and reperfusion**. In response to ischemia-induced increases in intracellular Ca^2+^, active Src kinase up-regulated ERK cascades through increment of Raf-1 phosphorylation at its active site (Tyr340/341). Meanwhile, Src kinase also contributes to ERK activation through increment of PP2A phosphorylation at its inactivation site (Tyr307) in cerebral ischemia. These two converse signaling pathways undergo crosstalk through ERK and play important roles in ERK/CREB and ERα cascades following cerebral ischemia.

The Raf/ERK pathway couples receptor tyrosine kinase (RTK) to cell fate decisions, such as growth, proliferation, migration, differentiation and survival [30]. It is well known that non-receptor tyrosine kinases, such as Src, can activate the ERK cascade [6,7]. Our previous studies have indicated that Src kinase can up-regulate the ERK cascade through direct phosphorylation of Raf at Tyr340/Tyr341 in response to ischemic stroke [15]. Here, a novel mechanism was identified whereby Src kinase induces the ERK pathway in a PP2A-dependent manner in rat hippocampus following ischemia (Figure 5).

PP2A is a Ser/Thr-specific phosphatase capable of dephosphorylating and inactivating ERK [9,10,29]. Induction of Src results in inactivation of PP2A resulting in up-regulation of ERK activity in cerebral ischemia. Several lines of evidence support the role of PP2A in regulation of the Src/ERK pathway. First, cerebral ischemia results in sustained activation of Src kinase after 6 h reperfusion post-ischemia, accompanied by continuous phosphorylation of Tyr307 and inhibition of PP2A. Second, SU6656, an effective Src inhibitor, prevents PP2A phosphorylation resulting in up-regulation of PP2A activity. Third, cantharidin is a specific inhibitor of PP2A, which has little effect on PP1. Treatment with cantharidin abrogates the effects of the Src inhibitor, SU6656, allowing for upregulation of ERK activity following ischemia. These results indicate that Src upregulation of the ERK pathway in ischemic neurons requires inhibition of PP2A.

Src-induced phosphorylation and inactivation of PP2A was thought to be closely associated with intracellular calcium signaling [12]. In rat hippocampal neurons, the Src/ERK cascade is dependent on calcium influx elicited by upregulation of ion channels like NMDA receptor and IP3 receptor. In addition, inhibition of ion channels can inhibit Src and ERK activity after cerebral ischemia [2,4,31]. Our previous studies have also suggested that Src kinase can up-regulate the Raf/ERK cascade directly in a calcium-dependent manner following ischemia stroke [6]. Apparently, Src can activate the ERK cascade via coordinated activation of protein kinases and inactivation of protein phosphatases in a calcium-dependent manner.

ERK exert their function through up-regulation of nuclear transcription factors resulting in changes in gene expression [16]. Our present study indicates that there were no changes in subcellular localization of total protein levels of ERK in response to ischemic stimuli. Cerebral ischemia induced an increase in ERK phosphorylation and activity in membrane, cytoplasma, and nucleus in hippocampal neurons. Activated ERK in the nucleus is sufficient to target its intranuclear substrates like CREB and ERα. As transcription factors, CREB and ERα are localized primarily in the nucleus of rat hippocampal neurons and their activities are negatively regulated by PP2A. Therefore, CREB and ERα share similar mechanisms as downstream molecules of ERK, and are modulated by Src kinase through a complex signalling network dependent on PP2A inactivation. CREB and ERα are thought to be protective factors in the nucleus [20,21]. Upregulation of intranuclear ERK/CREB and ERα pathway is involved in neuroprotective gene expression in response to Src signals following cerebral ischemia [32].

## Conclusion

Activation of Src kinase in neurons during cerebral ischemia induces a complex signaling network leading to activation of ERK. In response to ischemia-induced upregulation of Src kinase, Raf, a positive regulator of the ERK signaling cascade, is activated. In addition induction of Src kinase activity also leads to inhibition of PP2A, a negative regulator of ERK activity. Activation of ERK through induction of Raf activity and inhibition of PP2A leads to activation of CREB and ERα, transcription factors, which is involved in neuronal protection from ischemic stress.

## Methods

### Transient cerebral ischemia

Adult male Sprague-Dawley rats weighing approximately 250 g (Shanghai Laboratory Animal Center, SLAC, China) were housed under natural conditions (12-h light/12-h dark cycle from 07:00 to 18:00 h) at a constant temperature of 25°C with food and water *ad libitum*. All animal surgery was performed in accordance with the Institutional Animal Care and Use Committee and conformed to international guidelines on the ethical use of animals (Guide for the Care and Use of Laboratory Animal, 1996). Before surgery, the animals were deprived of food and water overnight. Experimental rats were anesthetized with 20% chloral hydrate (300 mg/kg, i.p.) and subjected to four-vessel occlusion (4VO) according to the method of Pulsinelli *et al*., 1982. Briefly, in sterile conditions while animals were under deep anaesthesia, the bilateral vertebral arteries were occluded permanently by electrocauterization and the common carotid arteries were separated from connective tissue and nerves and marked with surgical thread. On the following day, ischemia was induced by bilateral occlusion of the common carotid arteries for 10 min using aneurysm clips. To minimize the experimental variations, all rats used had to satisfy the following criteria: (1) complete flat electroencephalogram throughout the duration of carotid occlusion, (2) maintenance of dilated pupils and an absence of cornea reflex during ischemia, (3) rigor of the extremities and vertebral column. Sham operated control rats received the same treatment without carotid artery occlusion. Body temperature was monitored using a rectal probe and maintained at approximately 37°C using a heating pad until the animal had fully recovered from anesthesia.

### I.c.v. infusion and administration of inhibitors

Ten microliter microsyringes were used for injections. Polyethylene tubing was used to attach injection cannula to the microsyringe. A burr hole was drilled in the skull measured from bregma (0.8 mm posterior, 1.5 mm lateral and 3.5 mm deep) for administration of chemicals or vehicle before ischemia. Three microliters of cantharidin (Ct, 25 μg/μl; Biomol, Plymouth Meeting, PA) and SU6656 (SU, 5 μM; Calbiochem, La Jolla, CA, USA) (in DMSO) were administered, either individually or in combination, into the cerebral ventricle (i.c.v.). After the injection, the injector was retained in place for an additional 5 min in order to reduce any possible backflow of the liquid along with the injection void. Occlusion occurred 20 min post-injection.

### Subcellular fractionation and sample preparation

Rats were euthanized by decapitation at various time points: 10 min after ischemia or 10 min, 1 h, 6 h, or 24 h post-reperfusion. The hippocampi were quickly removed on ice in a cold room [6,33] and the separated brain regions were homogenized in 1:10 (w/v) ice-cold homogenization buffer A (HEPES 50, pH 7.4, KCl 100, Na_3_VO_4 _1, NaF 50, and PMSF 1 mM) supplemented with 1% mammalian protease inhibitor cocktail (Sigma-Aldrich Co., St. Louis, MO). Cytoplasmic and membrane proteins were extracted by centrifugation at 800 × *g *for 20 min at 4°C. Following centrifugation, the supernatant was transferred into the fresh tubes, which containing the membrane and cytoplasmic proteins (M and C). The resulting pellet was resuspended in homogenization buffer B (buffer A, 1 mM DTT, 1% cocktail and 10% NP-40), kept on ice for 30 min, intensely shaken for 15 min, and then centrifuged at 14,000 × g for 20 min at 4°C. The supernatant contains the nuclear protein (N). All of the supernatant was extracted and then stored at -80°C until assayed. The protein concentrations of the extracts were determined according to the Bradford assay protocol using bovine serum albumin (BSA) as a standard (Bradford, 1976).

### Western blot analysis

Equivalent amounts of protein lysates were resolved by 10% SDS-PAGE, transferred to nitrocellulose membrane, incubated in a solution of 3% BSA-Block/TBST for 2 h at room temperature, and then incubated with primary antibody in 1% BSA with shaking at 4°C overnight. Following three 5–10 min washes in TBST, the membranes were incubated with secondary antibody conjugated to horseradish peroxidase in 1% BSA at room temperature for 2 h. Following the incubation, membranes were given three 10-min washes with TBST, and then developed using the Pierce SuperSignal^® ^chemiluminescence kit (Pierce, Rockford, IL). The primary antibodies used were as follows: mouse anti-phospho-ERK (T202/Y204), rabbit anti-ERK, rabbit anti-Src, rabbit anti-PP2A C subunit, rabbit anti-phospho-CREB (Ser133), and rabbit anti-CREB purchased from Cell Signaling Technology (Beverly, MA); rabbit anti-phospho-ERα (Ser118) and rabbit anti-ERα purchased from Santa Cruz Biotechnology (Santa Cruz, CA); rabbit anti-phospho- PP2A (Y307) purchased from Abcom Biotechnology (Cambridge, UK), rabbit anti-phospho-Src (Tyr527) purchased from Sigma (St. Louis, MO); β-actin purchased from Boster Biotechnology (WuHan, HB, China). Blots were then washed four times for 15 min and visualized using enhanced chemiluminescence (ECL; Amersham Biosciences, Piscataway, NJ).

### PP2A activity assay

PP2A activity was measured with a molybdate dye-based phosphatase assay kit (Promega, Madison, WI). Assays were performed according to the manufacturer's protocol. Briefly, the samples were homogenized in lysis buffer (Tris-HCl 50, pH 7.4, EDTA 0.1, DTT 1, PMSF 0.1 mM, 1% Triton X-100 and 1% protease inhibitor cocktail) in ice water. Samples were filtered through Sephadex G-25 columns to remove free phosphate. The samples were then added to a reaction premix which contained phosphopeptide substrate, 5 × PP2A reaction buffer (250 mM imidazole, pH 7.2, 1 mM EGTA, 0.1% β-mercaptoethanol, and 0.5 mg/ml BSA) and storage buffer in 96-well plates. The reactions were terminated by adding a molybdate dye/additive mixture and incubating at 37°C for 30 min. Finally, phosphotase activity in the samples were measured using a spectrophotometer (Molecular Devices, Sunnyvale, CA) at 630 nm wavelength.

### Data analysis

In each case, for semi-quantitative analysis, the data from at least four animals are expressed as the mean ± standard deviation. Statistical analysis was conducted using an analysis of variance (ANOVA) followed by the Newman-Keuls test. Comparisons between two groups were performed using a t-test. *P *values less than 0.05 were considered significant.

## Abbreviations

ERK: Extracellular signal – regulated kinase; PP2A: Protein phosphotase 2A; CREB: cyclic AMP response element-binding protein; ERα: estrogen receptor α; MAPK: mitogen-activated protein kinase; NMDA: N-methyl-D-aspartic acid; L-VGCC: L-type voltage-gated calcium channels; IP3: Inositol 1,4,5-Triphosphate; PTK: protein tyrosine kinase; SH: Src homology; SU: SU6656; Ct: Cantharidin; 4VO: four-vessel occlusion; i.c.v.: intracerebral ventricular; BSA: bovine serum albumin; PAGE: polyacrylamide gel electrophoresis; Veh: vehicle.

## Authors' contributions

XH, XW carried out the 4-VO model and sample preparation, participated in the Western blot analysis and drafted the manuscript. JX participated in PP2A activity assay. JZ participated in the I.c.v. infusion. XH, JG conceived of the study, and participated in its design and coordination. All authors read and approved the final manuscript.
